# Candesartan, rather than losartan, improves motor dysfunction in thioacetamide-induced chronic liver failure in rats

**DOI:** 10.1590/1414-431X20176665

**Published:** 2017-09-21

**Authors:** H.A. Murad, Z.J. Gazzaz, S.S. Ali, M.S. Ibraheem

**Affiliations:** 1 Department of Pharmacology, Faculty of Medicine, Rabigh, King Abdulaziz University, Jeddah, Saudi Arabia; 2 Department of Pharmacology, Faculty of Medicine, Ain Shams University, Cairo, Egypt; 3 Department of Medicine, Faculty of Medicine, Rabigh, King Abdulaziz University, Jeddah, Saudi Arabia; 4 Department of Anatomy, Faculty of Medicine, King Abdulaziz University, Jeddah, Saudi Arabia; 5 Department of Microbiology, Faculty of Medicine, Rabigh, King Abdulaziz University, Jeddah, Saudi Arabia

**Keywords:** Candesartan, Hepatic fibrosis, Liver failure, Losartan, Motor dysfunction, Thioacetamide

## Abstract

Minimal hepatic encephalopathy is more common than the acute syndrome. Losartan, the first angiotensin-II receptor blocker (ARB), and candesartan, another widely-used ARB, have protected against developing fibrogenesis, but there is no clear data about their curative antifibrotic effects. The current study was designed to examine their effects in an already-established model of hepatic fibrosis and also their effects on the associated motor dysfunction. Low-grade chronic liver failure (CLF) was induced in 3-month old Sprague-Dawley male rats using thioacetamide (TAA, 50 mg·kg^−1^·day^−1^) intraperitoneally for 2 weeks. The TAA-CLF rats were randomly divided into five groups (n=8) treated orally for 14 days (mg·kg^−1^·day^−1^) as follows: TAA (distilled water), losartan (5 and 10 mg/kg), and candesartan (0.1 and 0.3 mg/kg). Rats were tested for rotarod and open-field tests. Serum and hepatic biochemical markers, and hepatic histopathological changes were evaluated by H&E and Masson's staining. The TAA-CLF rats showed significant increases of hepatic malondialdehyde, hepatic expression of tumor necrosis factor-α (TNF-α), and serum ammonia, alanine aminotransferase, γ-glutamyl transferase, TNF-α, and malondialdehyde levels as well as significant decreases of hepatic and serum glutathione levels. All treatments significantly reversed these changes. The histopathological changes were moderate in losartan-5 and candesartan-0.1 groups and mild in losartan-10 and candesartan-0.3 groups. Only candesartan significantly improved TAA-induced motor dysfunction. In conclusion, therapeutic antifibrotic effects of losartan and candesartan in thioacetamide-induced hepatic fibrosis in rats are possibly through angiotensin-II receptor blocking, antioxidant, and anti-inflammatory activities. Improved motor dysfunction by candesartan could be attributed to better brain penetration and slower “off-rate” from angiotensin-II receptors. Clinical trials are recommended.

## Introduction

Acute hepatic encephalopathy (HE) affects 30–45% of patients with severe liver disease while minimal hepatic encephalopathy (MHE) affects 20–60% of patients with liver disease (1). MHE resulting from common causes such as viral hepatitis and drug-induced hepatotoxicity is more common than acute HE (2). Due to disturbed metabolic functions in the diseased liver, HE is manifested by multiple abnormalities including motor dysfunctions (3). Many studies have confirmed that the renin-angiotensin system (RAS) plays a fundamental role in pathophysiology of liver fibrosis (4,5). In patients with chronic liver diseases, activation of the hepatic RAS generates angiotensin-II, which via its receptors activates the hepatic stellate cells and upregulates expression of transforming growth factor β1 (TGF-β1). Consequently, the angiotensin converting enzyme inhibitors (ACEIs) and the angiotensin receptor blockers (ARBs) attenuate progression of fibrosis in both animal and human studies (6).

Losartan, the first discovered ARB, alleviated carbon tetrachloride-induced hepatic fibrosis in Sprague-Dawley rats. It significantly decreased serum aspartate and alanine aminotransferase, and hepatic hydroxyproline. It also inhibited histopathological fibrotic changes and decreased tumor necrosis factor-α (TNF-α) and TGF-β1 levels in culture supernatants of Kupffer cells (7). Through antagonizing angiotensin II, losartan decreased the expression of the angiotensin II-activated NADPH oxidase in the inflammatory areas in the liver and consequently suppressed the oxidative stress (8). Candesartan, another widely used ARB, significantly attenuated hepatic fibrosis development in rats. It suppressed hepatic hydroxyproline, hepatic TGF-β1 protein and mRNA levels, and serum fibrosis markers (9). ARBs differ in their pharmacological characteristics. Candesartan, being lipophilic, can easily penetrate the blood brain barrier (BBB) while losartan, one of the least lipophilic ARBs, cannot. However, this information is still inconclusive (10).

Hundreds of substances were found useful in preventing development of experimental hepatic fibrosis, i.e. prophylactic effect, while there is no data about their effects in models of established fibrosis, i.e. therapeutic effects. Thus, it would be “more sensible if drug candidates are tested in models of established fibrosis to confirm their hepatoprotective or anti-fibrotic effects” (11). Consequently, the current study was designed to examine effects of losartan and candesartan in a pre-established model of fibrosis in rats. We hypothesized that candesartan would improve the motor dysfunction in thioacetamide-induced chronic liver failure in a model of established hepatic fibrosis in rats while losartan would not. We assumed that this effect is molecule-specific, i.e., it is a drug and not a class effect.

## Material and Methods

### Animals and drugs

The study protocol was approved by the King Abdulaziz University Research Ethics Committee and it adhered to the International Guidelines for the Care and Use of Laboratory Animals. Sprague-Dawley male rats aged 3 months and weighing 250–300 g were obtained from King Fahd Research Center and housed in cages at 20°–22°C room temperature in a 12-h light-dark cycle. Food and water were available *ad libitum*. All drugs and chemicals were purchased from Sigma-Aldrich Corp. (USA), unless mentioned otherwise.

### Thioacetamide-induced chronic liver failure and treatment groups

Low-grade chronic liver failure (CLF) was induced in rats by injection of thioacetamide (TAA, 50 mg/kg) intraperitoneally once daily for two weeks while a control group was given saline solution for the same duration (12). The thioacetamide-induced CLF was used for neurobehavioral assessments in rats (13). This model of mild CLF resembles MHE in humans. The TAA-CLF rats were randomly divided into five groups (n=8) treated once daily for 14 days by gastric gavage as follows: distilled water (control group), 5 (L5) and 10 mg/kg (L10) losartan potassium groups (14) and 0.1 (C0.1) and 0.3 (C0.3) mg/kg candesartan cilexetil groups (15). At the end of treatment duration, rats were tested with the rotarod test and the open-field test for locomotor activity. Later, blood was collected and serum was separated and kept at –80°C for biochemical measurements. Rats were sacrificed by cervical dislocation and livers were isolated for biochemical and histopathological assessments as described below.

### Rotarod test

Rats were placed on a Rotarod apparatus (Ugo Basile, Italy) at a fixed speed (32 rpm). The fall-off latency (time spent until the rat fell off from the rotarod) was recorded. The maximum latency time was set at 180 s. The rotarod test demands competent motor coordination with constant attention, thus it is the most appropriate test for assessing psychomotor slowing in rodents (16).

### Open field test

The locomotor activity was recorded by placing rats in the Opto-Varimex apparatus (Columbus Instruments, USA). The distance travelled and numbers of the vertical movements were recorded for 30 min (17).

### Hepatic malondialdehyde (MDA) measurement

The hepatic MDA was assessed as a measure of lipid peroxidation by thiobarbituric acid (TBA) reaction according to Ohkawa et al. (18). The liver was homogenized in 1.15% potassium chloride buffer and centrifuged at 10,000 *g* for 10 min at 4^o^C. TBA (0.8%), hydrochloric acid (HCl, 0.25 N) and then trichloroacetic acid (10%) were mixed with the supernatants in different steps. The mixture was incubated for 20 min in boiling water and then centrifuged at 3000 *g* for 15 min at room temperature, and absorbance was read at 535 nm with a micro plate reader (Versa Max, Molecular Devices, USA).

### Hepatic glutathione (GSH) measurement

The hepatic GSH was measured by GSH assay kit (Cayman Chemical Company, USA). Ellman's Reagent (5,5'-dithiobis 2-nitrobenzoic acid, DTNB) was used according to Saha and Nandi (19). The assay involves the reduction of DTNB to a yellow product by sulfhydryl groups present in GSH. The liver was homogenized in one mL of 5% trichloroacetic acid and then centrifuged at 10,000 *g* for 30 min at 4°C. The GSH content in the supernatant was measured using the extinction coefficient of DTNB and corrected to the protein content in the sample.

### Serum measurements

After the behavior tests, blood was collected from each animal under light anesthesia by ether. Serum was separated and stored at –80°C until measurements were done using commercially available kits according to the manufacturer's protocol as follows: alanine aminotransferase (ALT), γ-glutamyl transferase (GGT), and ammonia using colorimetric kits (Sigma-Aldrich Corp.), TNF-α using an ELISA kit (Gentaur Molecular Products, USA) and reading at 450 nm using a microplate reader (Versa Max, Molecular Devices), and MDA and GSH using colorimetric kits (Cell Biolabs, USA).

### Histopathological examination

Samples of the liver were fixed in 10% phosphate-buffered formalin and then embedded in paraffin. Sections of 3–5 μm thickness were cut, stained with hematoxylin and eosin for routine examination and Masson's trichrome for collagen, and then examined by a light microscope. Photographs were taken using a Nikon (Japan) camera and the lesions were reported as mild, moderate or severe necrosis (20).

### Quantitative RT-PCR

After sacrifice of the rats, the liver samples were taken, immediately immersed in RNAlater solution, and stored at 4°C until RNA extraction. The total RNA was extracted from hepatic tissue (30 mg) using the RNeasy kit (Qiagen, Germany) according to the manufacturer protocol, dissolved in 30 µL nuclease-free distilled H_2_O, and stored at -20°C. The RT-PCR was done using 2 µL template in a 20-µL reaction which contained 0.25 μM of each primer and 12.5 µL Sybr Green Master Mix (Applied Biosystems, USA) (21). The primers were TNF-α forward: ACT GAA CTT CGG GGT GAT TG, and reverse: GCT TGG TGG TTT GCT ACG AC. Each run consisted of 50°C for 2 min and 95°C for 10 min followed by 45 cycles of 95°C for 15 s, 60°C for 20 s, and 72°C for 60 s. Glyceraldehyde 3-phosphate dehydrogenase (GAPDH) was used as an internal control. The percentage calculated was based on normalization of TNF-α mRNA level against the GAPDH mRNA level in the same sample (22,23).

### Statistical analysis

The chi-square test was used for comparison of nominal data. Quantitative data are reported as means±SE and were analyzed using SPSS version 18 (USA). One-way ANOVA followed by the Tukey's multiple comparison *post hoc* test was used to assess differences among groups. P<0.05 was considered to be statistically significant.

## Results

### Serum measurements

**Figure 1.**
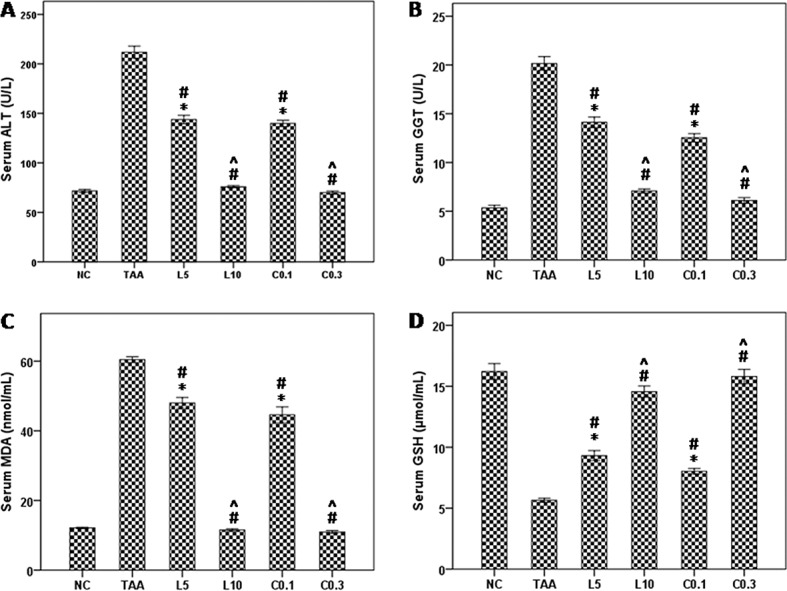
Effects of losartan and candesartan on the serum levels of: *A*, alanine aminotransferase (ALT), *B*, γ-glutamyl transferase (GGT), *C*, malondialdehyde (MDA), and *D*, glutathione (GSH) in thioacetamide (TAA)-induced chronic liver failure rats. Losartan (L5 and L10; 5 and 10 mg·kg^−1^·day^−1^) and candesartan (C0.1 and C0.3; 0.1 and 0.3 mg·kg^−1^·day^−1^) were given for 14 days by gastric gavage. Data are reported as means±SE, n=8. *P<0.05 *vs* normal control (NC); ^#^P<0.05 *vs* TAA group; ˆP<0.05 *vs* L5 and C0.1. One-way ANOVA followed by Tukey's multiple comparison *post hoc* test was used to assess differences among groups.

**Figure 2.**
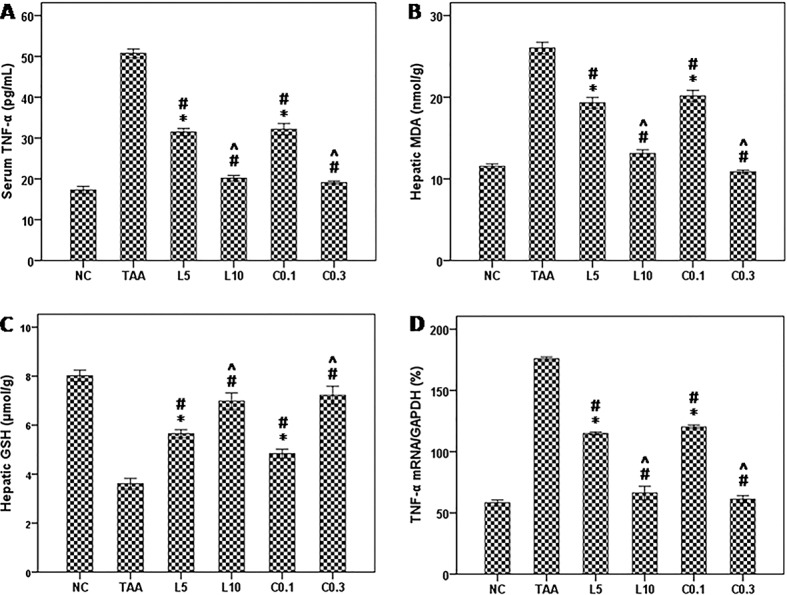
Effects of losartan and candesartan on the levels of: *A*, serum tumor necrosis factor-α (TNF-α), *B*, hepatic malondialdehyde (MDA), *C*, hepatic glutathione (GSH), and *D*, hepatic expression of TNF-α mRNA in thioacetamide (TAA)-induced chronic liver failure rats. Losartan (L5 and L10; 5 and 10 mg·kg^−1^·day^−1^) and candesartan (C0.1 and C0.3; 0.1 and 0.3 mg·kg^−1^·day^−1^) were given for 14 days by gastric gavage. Data are reported as means±SE, n=8. *P<0.05 *vs* normal control (NC); ^#^P<0.05 *vs* TAA group; ˆP<0.05 *vs* L5 and C0.1. One-way ANOVA followed by Tukey's multiple comparison *post hoc* test was used to assess differences among groups.

The TAA-CLF rats showed significant increases of the serum levels of ammonia, ALT, GGT, MDA, and TNF-α and a significant decrease of the serum GSH level. All treatments significantly reversed these TAA-induced changes. Both drugs showed dose-dependent effects. The L10 and C0.3 groups showed non-significant differences from the normal control and between each other (Table 1, Figures 1 and 2A).

**Table 1.** Effects of losartan and candesartan for 14 days by gastric gavage on the serum level of ammonia (µmol/mL) in thioacetamide (TAA)-induced chronic liver failure rats.

	NC	TAA	Losartan 5 (L5)	Losartan 10( L10)	Candesartan 0.1 (C0.1)	Candesartan 0.3 (C0.3)
Serum ammonia	0.23±0.01	0.43±0.02	0.35±0.02* ,#	0.26±0.02# ^,^ ˆ	0.34±0.01* ,#	0.24±0.02# ^,^ ˆ

Data are reported as means±SE, n=8. L5 and L10: 5 and 10 mg·kg^−1^·day^−1^ losartan; C0.1 and C0.3: 0.1 and 0.3 mg·kg^−1^·day^−1^ candesartan.

* P*<*0.05 *vs* normal control (NC);

# P*<*0.05 *vs* TAA group;

ˆ P*<*0.05 *vs* L5 and C0.1 (one-way ANOVA followed by Tukey's multiple comparison *post hoc* test.

### Hepatic measurements

The TAA-CLF rats showed significant increases of the hepatic MDA levels and a significant decrease of the hepatic GSH level. All treatments significantly reversed these TAA-induced changes. Both drugs showed dose-dependent effects. The L10 and C0.3 groups showed non-significant differences from the normal control and between each other (Figure 2B and C).

### Hepatic expression of TNF-α mRNA

The TAA-CLF rats showed a significant increase of the hepatic expression of TNF-α mRNA compared to the control rats. All treatments significantly reversed this TAA-induced change. Both drugs showed dose-dependent effects. The L10 and C0.3 groups showed non-significant differences from the normal control and between each other (Figure 2D).

### Rotarod and open field tests

**Figure 3.**
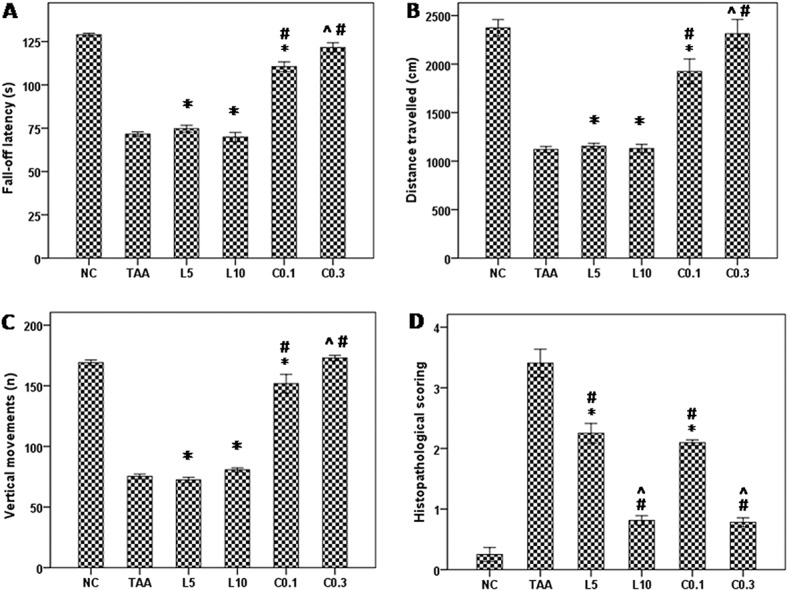
Effects of losartan and candesartan on the rotarod test (*A*), open field test (*B and C*), and hepatic histopathology (*D*) in thioacetamide (TAA)-induced chronic liver failure rats. Losartan (L5 and L10; 5 and 10 mg·kg^−1^·day^−1^), candesartan (C0.1 and C0.3; 0.1 and 0.3 mg·kg^−1^·day^−1^) were given for 14 days by gastric gavage. Data are reported as means±SE, n=8. *P<0.05 *vs* normal control (NC); ^#^P*<*0.05 *vs* TAA group; ˆP*<*0.05 *vs* C0.1. One-way ANOVA followed by Tukey's multiple comparison *post hoc* test was used to assess differences among groups in panels *A*, *B* and *C* and the chi-square test was used for comparison of scoring in panel *D*.

The TAA-CLF rats showed significant decreases of the fall-off latency in the rotarod test, and distance travelled and number of vertical movements in the open field test compared to the control rats. Candesartan significantly improved the TAA-induced motor dysfunction in a dose-dependent manner, in which the higher dose group showed non-significant difference from the normal control group. On the other hand, losartan failed to produce any improvement (Figure 3A-C).

### Histopathological examination

**Figure 4.**
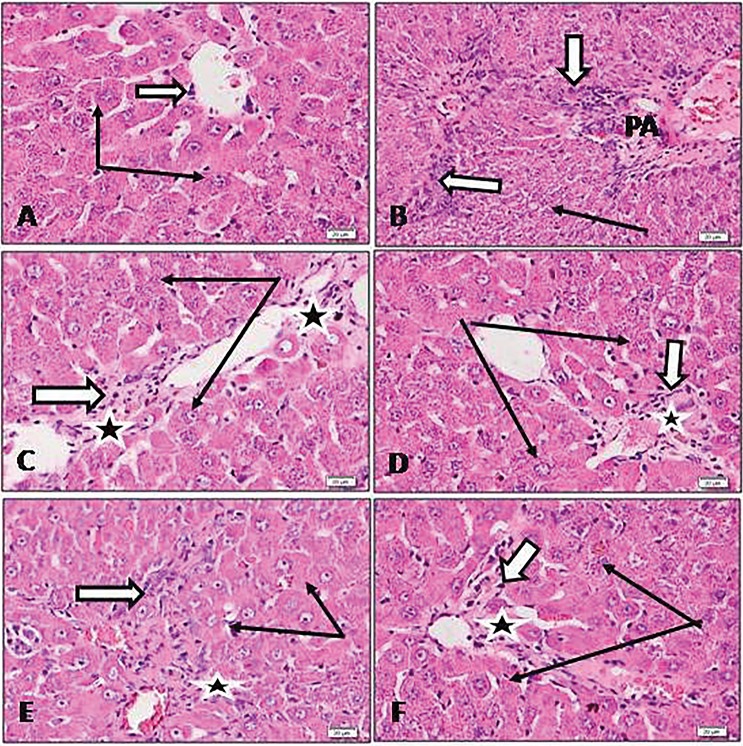
Sections of rat liver stained with H&E (×20). *A*, Normal control group showing normal hepatic lobulation and hepatocytes (black arrows). *B*, Thioacetamide group showing loss of normal architecture and early signs of hepatic fibrosis. There are strands of fibroblasts proliferation between lobules, lymphocytic infiltrate (stars), and degenerative changes in hepatocytes (black arrows). *C* and *E*, Lower doses of losartan and candesartan groups (5 and 0.1 mg/kg) showing potential improvement of fibrotic changes with moderate restoration of hepatic lobule architecture, moderate hepatocytes degeneration (black arrows), and less inflammatory infiltrate (stars). *D* and *F*, Higher doses of losartan and candesartan groups (10 and 0.3 mg/kg) showing marked improvement with minimal fibrotic changes, mild fibroblast proliferation, minimal inflammatory infiltrate. Most hepatocytes looked normal with minimal residual degenerative changes (black arrows). White arrows indicate newly proliferating fibroblasts.

The hepatic sections stained by H&E or Masson's trichrome showed the same pattern. The normal control rats showed normal ill-defined lobules, where the hepatocyte cell cord radiated from the central vein to the peripheral portal area that showed absence of any signs of fibrosis or inflammatory changes. The TAA-CLF rats showed chronic mild fibrosis manifested by development of fibrous strands bridging the portal areas and delineating liver lobules with disturbed architecture, collagen deposition, and congestion of the portal vessels. Hepatocytes showed moderate damage with inflammatory cell infiltration. The TAA-induced changes were graded as moderate in L5 and C0.1 and mild in L10 and C0.3 with images nearly similar to the normal control rats (Figures 3D, 4 and, 5).

**Figure 5.**
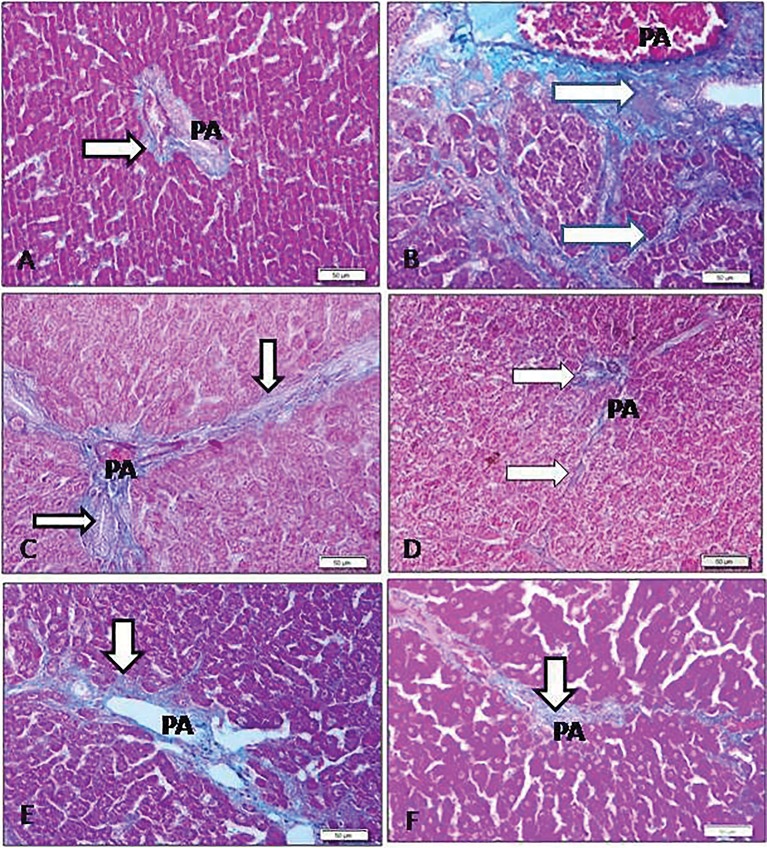
Sections of rat liver stained with Masson's trichrome (×600). *A*, Normal control group showing normal collagen distribution around portal area (PA). *B*, Thioacetamide group showing increased collagen deposition in the portal area (PA) and fibrous bridging between lobules (arrows). *C* and *E*, Lower doses of losartan and candesartan groups (5 and 0.1 mg/kg) showing potential improvement with decreased amount of collagen deposition at portal area (PA) and between lobules (arrows). *D* and *F*, Higher doses of losartan and candesartan groups (10 and 0.3 mg/kg) showing marked improvement with marked decrease of collagen deposition around portal area (PA), and absence of interlobular fibrosis (arrows). White arrows indicate newly proliferating fibroblasts.

## Discussion

In rats, candesartan significantly attenuated the development of carbon tetrachloride-induced hepatic fibrosis (24). In chronic hepatitis C patients candesartan in standard doses exerted an antifibrotic effect detected by measuring the following noninvasive fibrotic indexes: Forns index, the AST to platelet ratio index and FibroIndex” (25). Losartan attenuated development of liver fibrosis in animals (26). Also, in patients with chronic hepatitis C infection, losartan decreased fibrosis progression and expression of fibrogenic genes (8). In rats, TAA (*ip*) induced lipid peroxidation, glutathione S-transferase, and xanthine oxidase leading to formation of free radicals that cause oxidative damage, increases of hepatic damage markers and MDA, a decrease of hepatic GSH level, and liver necrosis (27). Losartan attenuated hepatic fibrosis partly through inhibition of xanthine oxidase because angiotensin-II activated endothelial xanthine oxidase causing oxidant stress (28). Fibrosis results in progressive organ dysfunction and finally failure. Galectin-3 is a profibrotic lectin protein and its inhibition could be a promising therapy for fibrosis (29). Treatments with galectin inhibitors reversed the TTA-induced fibrosis in the portal and central areas in rats and hence improved cirrhosis (30). Losartan relieved dystrophic epidermolysis bullosa at least partly due to reducing the increased level of galectin-3 (31), thus inhibition of galectin-3 could be an additional mechanism of losartan in treating fibrosis. *In vivo* studies showed that inhibition of TNF-α has antifibrotic effect in animals, however, the role of TNF-α in liver fibrosis, whether profibrotic or antifibrotic, is still controversial (32). Inhibition of RAS in liver could be an emerging therapeutic target for the treatment of hepatic fibrosis and thus candesartan and losartan, among other agents, are currently listed on <ClinicalTrials.gov> for the screening of their antifibrotic effects (33).

The TAA-CLF rats showed significant decline in riding time in the rotarod test due to motor slowing and dysfunction compared to control rats (12). Hyperammonemia activates cerebellar astrocytes and microglia leading to neuroinflammation, which is associated with increased extracellular gamma amino butyric acid in the cerebellum causing motor in-coordination and impaired learning ability in the Y maze (34). This could be equivalent to the motor dysfunction in MHE patients, which include subclinical motor slowing, impaired visual perception, impaired visuo-constructive ability, mild cognitive impairment, impaired ability to do memory tasks due to attention deficit, impaired performance in recognition tasks, and altered working memory (3).

Administration of candesartan 5 h before traumatic brain injury improved motor skills on the rotarod in mice. Co-administration of peroxisome proliferator-activated receptor-gamma (PPARγ) antagonist significantly reduced this effect indicating that candesartan's neuroprotective effect occurs through AT1-receptor blocking and PPARγ activation (35). In male Wistar rats, oral candesartan (3 and 5 mg/kg) for three weeks improved the tardive dyskinesia induced by the typical antipsychotics possibly via antioxidant and anti-inflammatory effects (36). In contrast, treatment with subcutaneous losartan (10 mg·kg^−1^·day^−1^) for 2 weeks in male Wistar rats did not cause any difference in motor coordination (37). In ischemia reperfusion injury in rats, 1 week-pretreatment with candesartan (0.1 and 0.3 mg/kg), protected against cerebral ischemia indicating a neuroprotective effect. It significantly improved the neurobehavioral tests (locomotor activity and rotarod test), MDA, and oxidative damage (15).

Generally, the antagonistic activities of candesartan and losartan in plasma are almost equal, but the *in vivo* antagonistic effect of candesartan is greater. Candesartan has a slower “off-rate” from the AT1 receptor than that of losartan (38). Candesartan has maximal binding affinity to AT1 receptor subtype while losartan has minimal (39). In addition, candesartan is highly lipophilic with a high brain penetrating ability (10) and losartan is one of the least lipophilic ARBs and thus intravenous losartan (1, 3 or 10 mg/kg) partially penetrated BBB. It produced a dose-dependent inhibition of AT1 receptor subtype in brain structures both outside and within the BBB while it did not affect the nuclei that contain AT2 receptor subtype (40).

In conclusion, both candesartan and losartan exerted curative antifibrotic effects against thioacetamide-induced chronic liver failure in rats possibly through their angiotensin-II receptor blocking, antioxidant, and anti-inflammatory activities. Only candesartan improved the associated motor dysfunction possibly due to better brain penetration and slower “off-rate” from the angiotensin-II receptor suggesting a potential benefit in this respect. Clinical trials are recommended to prove or refute these experimental results.
